# Unravelling the Network of Nuclear Matrix Metalloproteinases for Targeted Drug Design

**DOI:** 10.3390/biology9120480

**Published:** 2020-12-19

**Authors:** Anastasia S. Frolova, Anastasiia I. Petushkova, Vladimir A. Makarov, Surinder M. Soond, Andrey A. Zamyatnin

**Affiliations:** 1 Institute of Molecular Medicine, Sechenov First Moscow State Medical University, 119991 Moscow, Russia; frolanasta@gmail.com (A.S.F.); asyapeti@gmail.com (A.I.P.); known.sir@yandex.ru (V.A.M.); surinder.soond@yandex.ru (S.M.S.); 2 Belozersky Institute of Physico-Chemical Biology, Lomonosov Moscow State University, 119992 Moscow, Russia; 3 Department of Biotechnology, Sirius University of Science and Technology, 1 Olympic Ave., 354340 Sochi, Russia

**Keywords:** matrix metalloproteinase, extracellular matrix, nuclei, cancer, apoptosis, immune response

## Abstract

**Simple Summary:**

Nuclear matrix metalloproteinases are emerging to have distinct functions in a number of pathological conditions and physiological processes. In this article, we review what progress has been made in this area of research and discuss their potential in being targeted for future therapeutic design.

**Abstract:**

Matrix metalloproteinases (MMPs) are zinc-dependent endopeptidases that are responsible for the degradation of a wide range of extracellular matrix proteins, which are involved in many cellular processes to ensure the normal development of tissues and organs. Overexpression of MMPs has been observed to facilitate cellular growth, migration, and metastasis of tumor cells during cancer progression. A growing number of these proteins are being found to exist in the nuclei of both healthy and tumor cells, thus highlighting their localization as having a genuine purpose in cellular homeostasis. The mechanism underlying nuclear transport and the effects of MMP nuclear translocation have not yet been fully elucidated. To date, nuclear MMPs appear to have a unique impact on cellular apoptosis and gene regulation, which can have effects on immune response and tumor progression, and thus present themselves as potential therapeutic targets in certain types of cancer or disease. Herein, we highlight and evaluate what progress has been made in this area of research, which clearly has some value as a specific and unique way of targeting the activity of nuclear matrix metalloproteinases within various cell types.

## 1. Introduction

Matrix metalloproteinases (MMPs) are involved in the degradation of extracellular matrix (ECM) proteins and regulate many fundamental cellular processes during normal bodily development and function [1]. As the ECM is important in maintaining the mechanical and biochemical properties of tissues, its normal turnover and regulation by MMPs is necessary to permit multiple functions, as in the cleavage and activation of signaling molecules, cellular differentiation, and wound healing [2,3,4,5,6,7]. However, dysregulation of MMP activity can contribute to a variety of pathological conditions. For example, some have been seen to modulate matrix erosion in osteoarthritis and rheumatoid arthritis, whereas expression of others is associated with the formation of atherosclerotic lesions, platelet aggregation, and the regulation of factors associated with cardiovascular disease [8,9]. Predominantly, the roles of MMPs in malignant tumor initiation, metastasis, and angiogenesis have received the greatest attention and which have highlighted them as good potential therapeutic targets for the treatment of certain types of cancer [9].

To date, 26 human MMP proteins have been identified, which belong to the M10 family of metallo-endopeptidases [10]. Based on substrate specificity, MMPs can be further categorized into collagenases (MMP-1, MMP-8, MMP-13, and MMP-18), gelatinases (MMP-2 and MMP-9), stromelysins (MMP-3, MMP-10, MMP-11, and MMP-17), matrilysins (MMP-7 and MMP-26), membrane-type MMPs (MMP-14, MMP-15, MMP-16, MMP-17, MMP-24, and MMP-25), and others (MMP-12, MMP-19, MMP-20, MMP-21, MMP-22, MMP-23, MMP-28, and MMP-29) [1]. Generally speaking, they are expressed by a broad range of cell types, such as epithelial cells, fibroblasts, osteoblasts, endothelial cells, vascular smooth muscle, macrophages, neutrophils, lymphocytes, and cytotrophoblasts [1].

**Figure 1:**
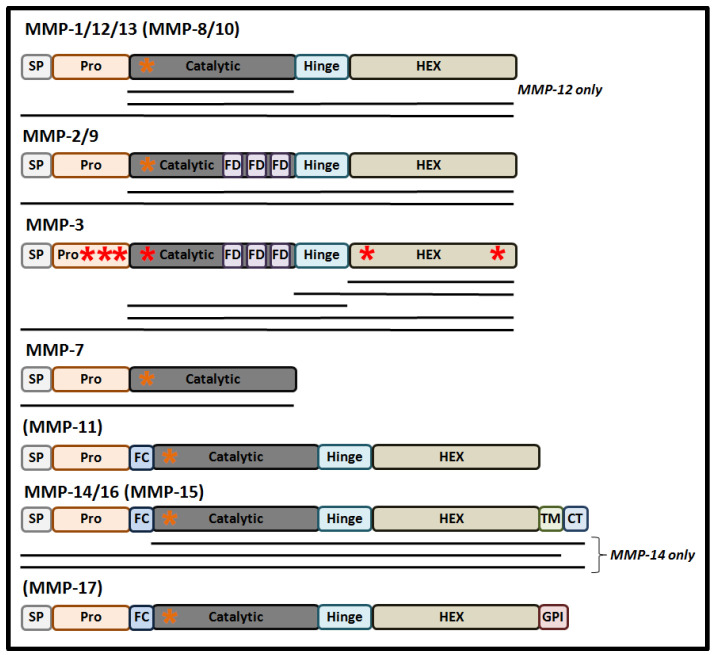
Domain structures of human matrix metalloproteinases (MMPs). Here the domain structures of nuclear MMPs (nMMPs) and MMPs, which have not been found in the nuclei but possess nuclear localization signals (NLSs) (placed in brackets), are presented. NLSs are indicated by the red asterisks (if their nuclear-trafficking properties have been proven experimentally) or by the orange asterisks (if the NLS has been identified by bioinformatics alone). Horizontal lines indicate the isoforms of MMPs, which have been found in nuclei. SP, signal peptide; Pro, pro-domain; FC, furin cleavage site; FD, fibronectin domain; HEX, hemopexin-like domain; TM, trans-membrane domain; CT, cytoplasmic tail; GPI, glycosylphosphatidylinositol.

Structurally, MMPs share a common protein domain structure (Figure 1). For most MMPs, the main components are a signal peptide (that directs synthesized protein into the secretory pathway), a highly conserved amino-terminal pro-domain, a catalytic domain that contains a zinc ion binding site, a linker domain, and a carboxyl-terminal hemopexin-like domain (HEX), that determines substrate specificity and localization and contributes to the enzymatic activity of MMPs [11].

These proteases are synthesized in the form of pre-pro-MMPs, with their enzymic activation occurring through the process of maturation as the proteins progress through the secretory pathway [1]. The first step of maturation is removal of the secretory signal peptide following the course of protein translation, giving rise to an inactive pro-MMP in which the inhibition of the catalytic site occurs through its resident Zn^2+^ ion binding a cysteine residue within the “cysteine switch” motif (PRCGXPD) present in the pro-domain [12]. Activation of the pro-MMP may occur in a variety of different ways, arising in a number of MMP forms containing the full-length pro-domain, a processed form of the pro-domain, and in MMPs lacking the pro-domain. In the former two MMP derivatives, conformational changes caused by mechanical or chaotropic agents can lead to the disruption of the Zn^2+^-Cys interaction resulting in pro-MMP activation in the absence of pro-domain cleavage [13]. Moreover, processed cleavage and removal of the pro-domain, by plasmin or trypsin, can mediate a conformation change of the protease resulting in full activation of the MMP intermediate [13]. Normally, full cleavage of the pro-domain is either mediated by the furin pro-protein convertase in the trans-Golgi network, auto-catalytically, or by other MMPs at the cell’s surface, either within the ECM or the nucleus [12,14,15]. The activity of MMPs can also be regulated by post-translational modifications, such as glycosylation, phosphorylation, and by glycosaminoglycans (GAGs). For example, glycosylation can stabilize a complex between MMP-14, TIMP2, and pro-MMP-2 as a step necessary for the cell-surface activation of MMP-2 [16]. Alternatively, glycosylation can promote MMP-9 secretion and activation, while also stabilizing the formation of MMP-17 dimers [17]. As an important step for the classical mode of MMP activation, a number of recent studies have also reported that some MMPs are also responsive to redox-mediated activation [18].

The tissue inhibitors of metalloproteinase (TIMPs) have also gained significant importance over the years based on their developmental role in normal tissue homeostasis and disease progression and their abilities to modulate MMP protease activity [19]. Four TIMPs (TIMPs 1-4) have been identified, and their mechanisms of MMP inhibition have been established through a number of structural studies. Residues 1–4 of the TIMP-1 amino-terminal domain interact with the primed side of the MMP binding pocket, where Cys-1 can coordinately bind the catalytic site Zn^2+^ ion. Simultaneously, five residues (spanning amino acids 66–70) from TIMP-1 can occupy the non-primed site [20]. These potential modes of binding were also shown to be highly conserved among TIMP-2, TIMP-3, and TIMP-4 [20,21]. Biologically, elevated TIMP expression levels have been shown to contribute to enhanced ECM accumulation and deposition, while reduced TIMP expression leads to enhanced matrix proteolysis, thus highlighting their importance in modulating ECM dynamics and plasticity [1,22]. TIMPs can also form non-inhibitory pro-MMP/TIMP/MT-MMP complexes, as in the instance of TIMP-2 complexing with MMP-14 and which can activate pro-MMP-2 in human fibrosarcoma, breast, and melanoma cell lines [16]. While TIMPs are generally found within the ECM, a number of studies have demonstrated that they may also reside in the nucleus of cells, as seen for TIMP-1 [23,24,25].

Over the years, matrix metalloproteinases have been pursued as good targets for therapeutic development [9], and have the potential to be targeted at several levels of their synthesis and maturation, the proposed stages of which include inhibition at the transcriptional level, during zymogen activation, and at the level of substrate catalysis by the active enzyme [9]. At the moment, there are MMP-directed targeted strategies coming into fruition for the treatment of inflammation, heart disease, lung diseases, and ischemic stroke [26,27,28,29,30]. Simultaneously, the search for more specific and better MMP inhibitors is still ongoing, driven by limited options for targeting specific MMPs within a clinical setting [31]. Consequently, novel strategies embodying greater specificity and efficacy have taken on a greater priority in targeting MMPs.

Over the last ten years, the nuclear localization of MMPs (nMMPs) has been an increasingly reported phenomenon, which has been observed in high-grade tumors, correlated with tumor volume, and in some instances has been associated with poor prognosis in a number of disease types (Table 1) [32,33,34,35,36]. Collectively, such findings suggest an important functional role for nuclear MMPs and that such a localization effect does have biological and clinical significance. In support of this, it is interesting to note that nuclear localization has been reported for other ECM proteases as well. For example, nuclear cathepsins L and D have been reported to exhibit biological effects which can contribute to tumor progression [37,38,39]. Collectively, the localization of such proteases have the potential to activate or deactivate transcription factors, regulate chromatin remodeling, apoptosis, alter the structural elements of the nuclear matrix, and participate in molecular events that lead to cell proliferation and carcinogenesis [40,41,42,43].

What signaling cues cause MMPs to be directed to the nucleus still largely remains unknown, with a number of mechanisms being proposed, which stem from environmental factors to cellular metabolism [44]. Nevertheless, for several MMPs, researchers have been able to propose some molecular mechanisms responsible for nuclear MMP translocation [45,46,47,48,49,50,51,52].

In this review article, we highlight the increasing emergence of nMMPs, while outlining their biological significance to highlight how these distinct sub-sets of proteases may have good targeting potential in diseases such as cancer.

## 2. Mechanistic Regulation of Nuclear MMPs

One of the commonest ways to deliver proteins from the cytoplasm to the nucleus is through the process of receptor-mediated nuclear shuttling and due (in large part) to proteins possessing a nuclear localization sequence (NLS) [53]. Here, importins α and β recognize and bind the NLS to form an importin-cargo complex, which can bind the nuclear pore complex, and facilitate the translocation of protein cargo from the cytoplasm into the nucleus [54]. Depending on its sequence and structure, the NLS can be sub-divided into two groups composed of the classical NLS and the proline-tyrosine (PY) NLS. Specific importin proteins are also involved in recognizing different types of NLS, which allows them to confer protein selectivity and regulate this mechanism with greater specificity [53].

**Figure 2:**
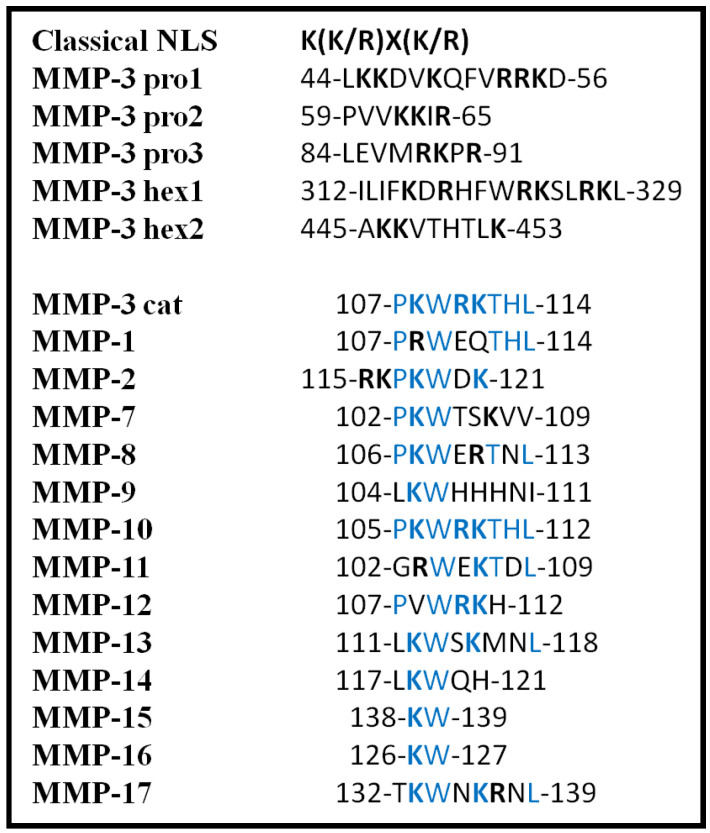
Nuclear localization sequences found in human MMP proteins. The consensus sequence for the classical NLS is indicated at the top of the figure. Three NLSs from the MMP-3 pro-domain (pro1-3) and two NLSs from the hemopexin-like domain (hex1-2) are highlighted. The NLS from the catalytic domain of MMP-3 is shown as a reference sequence, for comparison purposes with putative NLSs from the catalytic domains of other MMPs. Similar or identical sequences within the aligned NLSs are indicated with blue.

The classical NLS was originally thought to be involved in the nuclear translocation of MMP-2 when this protease was detected in the nucleus of rat cardiac myocyte cells for the first time [55]. This was confirmed upon scrutinizing the rat MMP-2 protein sequence, which revealed two small stretches of basic amino acids close to the C-terminal separated by a variable spacer [55]. Such sequences were also identified in the catalytic domain of MMP-3 [56]. The putative NLS (PKW**RK**TH) was identified and confirmed using the bioinformatics software, Protein Subcellular Localization Prediction Tool (PSORT, https://psort.hgc.jp/) [57], and validated upon the deletion of two positively-charged amino acids from this putative NLS, which led to a large decrease in the nuclear localization of the mutated proteins [56]. The same outcomes were observed after the substitution of these amino acids with uncharged amino acids as in the amino acid substitutions **R**110N and **K**111Q. For the first time in this field of research, such findings demonstrated a potential molecular mechanism for the nuclear translocation of MMP-3 [56]. Subsequently, Eguchi et al. (2008) identified five additional putative NLSs in MMP-3, of lysine- and arginine-rich sequences and which were found to be dispersed throughout all of the MMP-3 protein domains (Figure 2) [58]. Moreover, all of these NLSs were exclusively able to transport the MMP-3 protein into the nucleus. While such a study highlighted the existence of multiple NLSs and their dispensability, it also suggested that the post-translational modification of MMPs may “hide” primary NLSs in addition to exposing alternative NLSs and which may offer potential mechanisms that confer selectivity for the nuclear shuttling of some proteins. Functionally, nMMP-3 has also been shown to participate in the transcriptional regulation of *CTGF/CCN2* and *HSP* gene expression, where the presence (or absence) of each of the NLSs may contribute to regulating TG2, ERK, and IL-33 specific signaling pathways and responses [58,59,60].

The use of bioinformatic analyses have also helped to develop this area of research through identifying additional putative NLS sequences in other human MMPs protein sequences. Here, Abdukhakimova et al. (2016) identified a putative NLS within the catalytic domain of 14 MMPs, including the above-mentioned MMP-2 and MMP-3 proteins [61]. The sequences of MMPs were compared with experimentally validated NLSs from the catalytic domain of MMP-3 (of sequence PKWRKTH) [58] and most of the recovered NLSs contained two consensus residues, namely lysine and tryptophan (KW). The whole sequence was identified only in MMP-3 and MMP-10 and the authors also revealed the importance of the NLS in MMP-7 through it being evolutionary conserved throughout different species (Figure 2) [50,61].

Mechanistically, it has been proposed that endocytosis may also be responsible for the nuclear localization of MMPs. For example, in hepatocellular carcinoma cells, the amount of nuclear MMP-14 protein was increased in comparison to healthy liver cells, an event which enhanced the metastatic capacity of tumor cells [33]. Here, MMP-14 was jointly localized within the cytoplasm and perinuclear space and could interact with caveolin-1, thus implicating a specialized form of endocytic-protein trafficking that is fundamentally different to the use of nuclear transport receptors [54,62]. In support of this, caveolin-1 has been reported to drive and enrich the transport of proteins to the nucleus in human endothelial cells, as seen with caveolae regulating the intracellular protein trafficking of MMP-14 [63,64]. Such observations enforce the proposition that caveolin-1 participates in the nuclear translocation of MMP-14.

For the first time, nTIMPs were reported in human gingival fibroblasts in 1995 and in human breast carcinoma cell lines in 1999, prior to the discovery of nMMPs [23,24]. Subsequently, Gasche et al. (2001), reported gelatinolytic activity in the nuclei of mouse brain cells after ischemia-reperfusion, for which nMMPs were suggested to be responsible for [65]. Two years later, Si-Tayeb et al. reported the detection of nMMP-3 in human hepatocellular carcinoma cell line (HepG2) and the identification of a nuclear localization signal (NLS) within the structure of the protease [66]. Since then, the number of reported nMMPs has grown, with some MMP members being localized to the nuclei in a variety of different cells types, originating from normal tissues, cancers, infected cells, and in cells during disease progression (Table 1) [67,68,69,70,71]. For example, nMMP-2 was found in normal skin cells in the lower one-third of the epidermis, whereas in the tumor and pre-cancerous samples, it was predominantly in the upper layers of the skin suggesting that the protein may be expressed at the early stages of squamous cell carcinogenesis [72]. The expression of MMP-7 and MMP-16 were also found in the nuclei of basal and supra-basal cells of normal squamous epithelium and condyloma [73]. Alternatively, MMP-12 was detected in the nuclei of the virus-transfected cells and MMP-14 was reported as being present in the nuclei of macrophages, supporting the possible involvement of MMPs in the immune response [74].

The only nTIMP identified so far, nTIMP1, was found co-localized with nMMP-2 in endothelial cells and neurons, but no direct protein interactions or mechanism(s) for TIMPs translocation have been defined [25]. nTIMP1 inhibits nMMP-9, which was identified in neuronal cells, and exhibits insignificant or low levels of nMMP-9-derived gelatinase activity [80,81,87]. nTIMP1 was detected in gingival fibroblasts (in which nMMP-1 and nMMP-9 were later identified), and also in breast carcinoma cells, where nMMP-1 was also subsequently identified [23,24,76,77]. The mechanistic relationship shared between nMMPs and nTIMPs based upon them simultaneously residing in the nucleus have not been fully investigated in model systems, but immunofluorescence analysis has indicated nMMP/nTIMP co-localization within the nucleus of neural stem cells in Huntington’s disease (HD) can contribute to enhanced neurotoxicity. Here, TGF-β treatment enhanced nTIMP1 protein levels, which conferred neuroprotection in HD against toxicity associated with the aggregation of neurotoxic mutant huntingtin proteins [71]. From Table 1, it is interesting to note that co-localization of nTIMP1 with every nMMP reported so far was detected (although not exclusively) in malignant tissues and a variety of other cell types. Additionally, while extracellular MMPs are seen to promote epithelial-mesenchymal transition (EMT), tumor invasion, and metastasis, nMMPs are reported to be present in both epithelial and the resulting mesenchymal cells, which is suggestive of them fulfilling potentially unique and distinct intracellular functions during EMT [92,93].

In summary, it is becoming firmly established that MMPs and TIMPs have the capacity to translocate to the cell’s nucleus. While some of these depend on the presence of a classical NLS sequence (or a sequence derived from this), others are capable of this event through caveolin-dependent endocytosis. Unveiling the underlying molecular mechanisms for nMMP and nTIMP transport may lay down solid foundations for such mechanisms to be potentially targeted.

## 3. Nuclear MMPs as Regulators of Gene Expression

**Figure 3:**
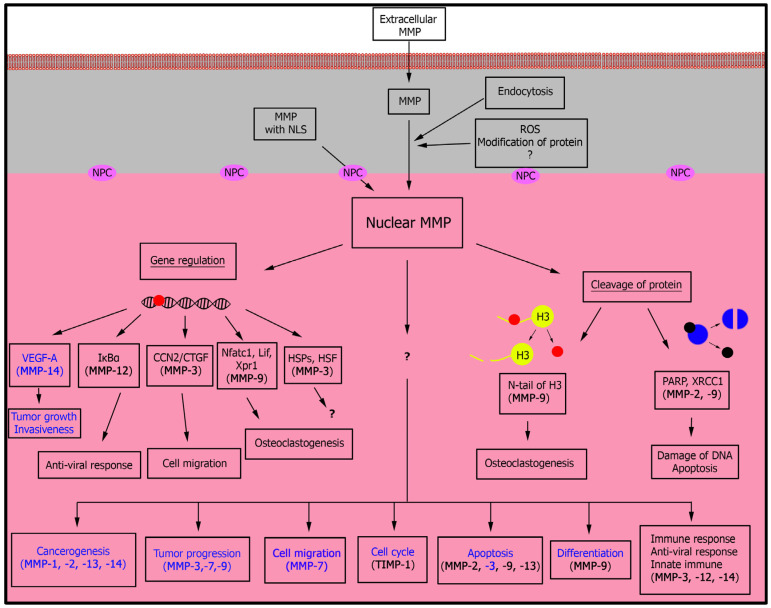
Role of nuclear MMPs within cells. Currently, only two mechanisms for the transport of MMPs into the cell’s nucleus are known, and which utilize the nuclear pore complex or endocytosis. Within the nucleus, MMPs can cleave nuclear proteins or regulate the transcription of various genes. Through these mechanisms, nMMPs can modulate a number of key biological processes within the cell. Participation of nMMPs in cancer cell progression is indicated in blue. NPC—nuclear pore complex; MMP—matrix metalloproteinase; NLS—nuclear localization signal.

Following the entry of MMPs into the nucleus, they have been shown to participate in a number of different processes, such as cell migration, proliferation, signaling pathways, tumor growth, and the immune response (Figure 3) [60,68,75,94,95,96]. As an area of research that has seen significant growth over the recent years, we outline a number of key publications that highlight the diverse biological effects that are modulated when the MMPs are resident within the nucleus.

Unlike the extracellular MMPs, nMMPs have access to genomic DNA and may therefore modulate gene expression events related to disease progression. For example, in the human bone osteosarcoma epithelial cells, MMP-2 was visualized by immunofluorescence methods in the nucleolus where it could interact with DNA associated with different regions of the ribosomal RNA genes, suggestive of its potential to regulate rRNA transcriptional initiation [97]. Here, the inhibition of MMP-2 activity by siRNA interference led to a slower cell proliferation rate in comparison to control cells.

In human breast carcinoma MCF7 cells, overexpression of MMP-14 significantly increased the transcriptional expression of vascular endothelial growth factor A (VEGF-A) [95]. Mechanistically, MMP-14-regulated VEGF-A expression could be suppressed through the treatment of cells with the Src-tyrosine kinase inhibitor PP2 [95], and whether this regulatory effect is direct or not is still to be revealed. Similarly, such findings also have great significance for the role of nMMP-14 in the promotion of tumor growth or invasiveness [98,99]. Here, nMMP-14 stimulated the expression of SMAD1 via TGF-β signaling [98]. Additionally, nMMP-14 suppressed the expression of Dickkopf-3 (DKK3) in human urothelial cell carcinoma tissue, which led to increased invasiveness of cells [99]. In support of this, while the localization of nMMP-14 was not the object of the investigation, the nuclear staining of MMP-14 has also been observed and reported independently by immunohistochemical methods in other studies [33,74].

Immunocytochemistry methods have also identified nMMP-3 [58]. It was shown that the HEX domain of nMMP-3 can interact with transcription enhancer dominant in chondrocytes (TRENDIC) within the connective tissue growth factor gene (*CTGF/CCN2*) promoter region and activate its transcription [58,60]. The proteins regulated by this promoter play an important role in proliferation, the formation of the extracellular matrix, angiogenesis, and cell migration. In human dental pulp, nuclear MMP-3 could also regulate the expression of CTGF/CCN2 proteins and the cellular migration capacity of cells through this pathway [60].

Although nuclear MMPs are known to participate in malignant tumor progression, they also have additional functions of importance that are related to normal cellular homeostasis. Here, the use of transcriptomic analyses revealed that overexpression of MMP-3 stimulated mRNA expression of heat shock proteins (HSPs), HSP70B, HSP72, HSP40, and HSP20. Several transcription factors that potentially interact with nMMP-3 were predicted and one of them, heat shock factor 1 (HSF1) was validated to co-activate the *HSP70B* gene promoter together with the nMMP-3 protein [59]. Of note, the HEX domain alone was sufficient to induce HSP70B expression. Other transcriptional factors that nMMP-3 may associate with include FOXO3, VDR, Ets-1, CULT1, TBP, and SP1. Since MMP-3-green fluorescent protein (GFP) was found in cellular chromatin fractions and soluble nuclear fractions of COS7 cells, in which nuclear markers chromobox protein CBX5/HP1α and histone-H3 were also detected, it was suggested that MMP-3 can also enter the cell’s nucleus to possibly modulate gene expression events [59].

Another protease, MMP-9, was found to contribute to osteoporosis, which is characterized by increased osteoclastogenesis and a decreased number of active osteoblasts (for bone formation). During osteoclastogenesis, nMMP-9 affected the expression of more than 67% of genes [86], normally expressed in primary osteoclast precursor cells, which included genes that regulate RANKL, AMPK, and VEGF signaling pathways. On a morphological level, inhibition of nMMP-9 enzyme activity led to reduced maturation of osteoclasts, compared with control cells. Using an alternative approach by incorporating ChiPac-seq technology, nMMP-9 was seen as being required for histone-H3 protein cleavage near the transcription start sites of the osteoclastogenic genes *Nfatc1*, *Lif*, *Xpr1*, and for their concurrent activation during osteoclastogenesis. Nuclear accumulation of MMP-9 was also confirmed by immunofluorescence microscopy [86].

Tetracyclines have antimicrobial activity, block bone deterioration and work as MMP-9 inhibitors [99]. Tetracycline analogs, minocycline, and tigecycline suppressed osteoclast formation by blocking nMMP-9-mediated proteolysis of the amino-terminal of histone-H3 protein [100]. Antibiotic treatments significantly reduced the differentiation of osteoclasts but did not affect the proliferation of pre-osteoblasts and osteoclast precursor cells. At the transcriptional level, both tetracycline analogs repressed RANKL-induced mRNA expression of the MMP-9-targeted genes *Nfatc1*, *Lif*, and *Xpr1*. Through using tigecycline and minocycline treatments on zebrafish larvae harboring an osteoporosis phenotype, the antibiotics reduced prednisolone-induced osteoporosis in a dose-dependent manner. Such antibiotics can therefore be effective as a treatment for osteoporosis through modulating nMMP-9 enzymatic activity towards the histone-H3 protein and its gene regulatory effects [100].

In summary, a number of novel functions for nuclear MMPs have emerged over the years, spanning the mechanistic entry of MMPs to the nucleus and their input into the regulation of gene expression in cell- and disease-context dependent manner. In particular, some nMMPs affect proliferation, migration, and invasion that contribute to tumor progression. These data also support the fact that nMMPs can influence cellular processes at the level of gene regulation, thus highlighting additional potential as targets in the treatment of cancer.

## 4. Nuclear MMPs as Regulators of Malignancy

Nuclear MMPs also mediate a malignant cell phenotype while contributing to cellular mobility and tumor progression. For example, nMMP-7 together with alternative reading frame (ARF) protein expression contributed to enhanced migration and metastasis of prostate cancer cells [68]. Knockdown of ARF expression in cancer cells decreased MMP-7 expression, but when ARF was over-expressed, MMP-7 accumulated in the nucleus where it could bind to the ARF protein. The molecular mechanisms responsible for this effect remain unclear, but the concurrent increase in these two proteins within the nucleus is correlated with malignancy of cancer cells and the combined targeting of ARF and MMP-7 may therefore have therapeutic value in the treatment of advanced prostate cancer.

The proteases MMP-3 and MMP-9 also contribute to tumor progression [35]. Significant expression of both non-proteolytically-active and proteolytically-active isoforms of these MMPs were found in metastatic cells derived from colon adenocarcinoma cell lines. After colon adenocarcinoma cells were injected into the abdominal walls of mice, primary tumors and metastatic tumors in lung tissues contained active nMMP-9 that had become localized within the nuclei of cells detected within the tumor-stromal area. The knock-down expression of MMP3 by siRNA during the latter experiments suppressed cancer cell migration, suggesting an important and significant contribution from nMMP-9 and MMP-3 during tumor invasiveness [35].

In summary, it is becoming increasingly apparent that intracellular MMPs can play multiple (yet significant) roles in tumor progression.

## 5. Nuclear MMPs and Oxidative Damage to DNA

The MMP proteases can process multiple DNA-interacting nuclear proteins during oxidative stress. For example, the accumulation and activation of MMPs were observed in the nuclei of ischemic cells after reperfusion [80,101]. Here, MMP-14 promoted the activation of the zymogens pro-MMP-2 and pro-MMP-9 within the nuclei of ischemic cells after reperfusion [80]. Catalytically active nMMP-2 and nMMP-9 have been shown to cleave the PARP-1 and XRCC1 proteins, which play an important role in DNA repair and caspase-independent cellular apoptosis [44,55]. It was reported that adenosine diphosphate could enhance the cleavage of PARP-1 by nMMP-2 and nMMP-9, through the PI3K/Akt/NF-κB and ERK1/2 signal transduction pathways [34]. Cleavage of PARP-1 and XRCC1 was also observed, which led to the accumulation of damaged DNA within the nuclei of ischemic brain cells after reperfusion. When rats were treated with the broad-spectrum MMP inhibitor BB1101, the cleavage of PARP-1 was significantly reduced and the amounts of XRCC1 protein were reported to increase. The use of such inhibitors in therapeutically targeting MMPs following cerebral ischemia-reperfusion injury is a good example of how nMMPs are being targeted for therapeutic purposes [81].

Other studies have also confirmed the accumulation of MMP-2 and MMP-9 in the nuclei upon ischemia treatment of cells. In the nuclei of mouse neurons deficient in superoxide dismutase (SOD1) and treated with ischemia-reperfusion, pro-MMP-2 and pro-MMP-9 protein levels were induced and activated. Active MMP-2 and MMP-9 are involved in the early-stage destruction of the blood-brain barrier, caused by oxidative stress during cerebral ischemia-reperfusion [65]. After an ischemic stroke in neurons and glial cells, protein MMP-9 was reported to be localized in the nucleus. The cells containing nMMP-9 also expressed activated caspase 3, which confirmed the link between the nuclear localization of MMP-9 and neuronal apoptosis in ischemic cells [102]. Similarly, the activated form of MMP-13 was also found in the nuclei of neurons as an early event following cerebral ischemia [15]. By subjecting the primary neural culture of rats to oxygen and glucose deprivation, Cuadrado et al. (2009) were able to demonstrate the nuclear translocation of MMP-13 in vitro.

Collectively, such important findings suggest that nMMPs may also fulfill a role in modulating the cell’s response to oxidative stress (in addition to disease progression) and that certain members of this family may also be directly be involved in the DNA damage response and caspase-dependent cell death.

## 6. Nuclear MMP and Apoptosis

Regulated cell death can occur in different ways in the form of apoptosis, necrosis, pyroptosis, and autophagy [103]. Whether the cell chooses the path of apoptosis or the path of survival depends on the ratio of pro- and anti-apoptotic factors, and in this context, MMPs have been found to modulate both pro-apoptotic [104] and pro-survival effects [105].

One of the environmental factors that can induce oxidative stress and apoptosis is cigarette smoke, which reportedly changes the expression levels of MMP-2, MMP-9, and TIMP-2 and their subcellular localization in pulmonary artery endothelial cells [79]. Here, the level of annexin V-positive/propidium iodide-negative cells significantly increased compared to untreated control cells indicative of enhanced apoptosis. Cells exposed to cigarette smoke contained PARP-1 protein fragments usually detected in apoptotic cells, including a high level of gelatinase activity. Since MMP-2 and MMP-9 were also observed to cleave PARP-1, these data suggest that cigarette smoke may induce apoptosis via MMP activation.

Alternatively, nMMP-1 may also have a pro-survival role. For example, MMP-1 is co-localized with mitochondria and the nucleus in normal glial Muller cells [75], but during staurosporine-induced apoptosis, MMP-1 expression changes and localizes to perinuclear mitochondrial clusters and around fragmented nuclei. Inhibition of MMP-1 activity led to lamin degradation, caspases activation, and apoptosis.

Nuclear MMP-3 expression in HepG2 and liver myofibroblast cells could also affect their rate of apoptosis [56]. When Chinese hamster ovary cells were transfected by a plasmid encoding an EFGP/active MMP-3 fusion protein, it principally localized in the nuclei. Using an antibody against activated caspase 3, it was determined that cells transfected with EGFP/active MMP-3 had higher apoptotic levels compared with untransfected cells. This effect was enhanced in cells where MMP-3 was present within the nucleus. Moreover, expression of a catalytically-inactive form of MMP-3 or inhibition of wild type MMP-3 in the presence of a broad-spectrum MMP inhibitor GM6001, led to a reduction in apoptotic cells. Such findings suggest another important biological effect for active nMMP-3 in apoptosis regulation.

Collectively, nuclear MMPs can have the effects of enhancing or decreasing apoptosis of cells. As described above, there is a clear relationship between nuclear MMP-2, MMP-3, MMP-9, and activation of apoptosis. Conversely, nMMP-1 can block the pathway of apoptosis. Such findings have been defined for a limited number of the MMP family members and clearly further developments in this area of research are warranted based on the importance of nMMPs and their contribution to disease progression [106].

## 7. Nuclear MMPs in Immune and Anti-Viral Responses

During inflammation, the expression profiles and activity of a wide range of proteases is increased. These include serine proteinases, such as granzymes, neutrophilic elastases, cathepsin G, and proteinase 3 [107]. Some of these proteases can modulate inflammation and the immune response via regulation of cytokines and chemokines [108]. For example, innate immunity is regulated by MMP-25, which is preferentially produced by leukocyte cells. While MMP-25-deficient mice were viable, they had defects in their innate immune system through having high sensitivity to bacterial lipopolysaccharide, hypergammaglobulinemia, and showed decreased secretion of the pro-inflammatory molecule COX2 [109]. In macrophages, nMMP-14 was observed to participate in the regulation of inflammation, the immune response, or anti-viral and innate immunity. Mechanistically, nMMP-14 can trigger expression and activate phosphoinositide 3-kinase δ-Akt-GSK3β signal cascade and modulate the Mi-2/NuRD nucleosome remodeling complex [74].

The immune system is also modulated by MMPs regulating the transcription of genes involved in anti-viral immunity. For example, macrophages can release MMP-12 during a viral infection, which can also enter infected cells and be translocated to the nucleus, presumably through endocytosis and lipid-dependent trafficking [95]. Through its catalytic domain, nMMP-12 can bind the polyA-rich regions within the promoter region of the *IκBα* encoding gene, and induce its transcriptional expression [70,89], which upregulates the secretion of interferon-alpha (IFN-α) [70]. However, in the absence of MMP-12 expression (in a knockout mouse model), in mice infected with Coxsackie B type B2 virus, unsecreted IFN-α protein remained within pancreatic, heart, and hepatocyte cells and the mice succumbed to the lethal effects of viral infection. Such effects on IFN- α expression could be reversed upon the artificial expression of MMP-12 in MMP-12^−/−^ fibroblast cells in vitro [70]. Moreover, nMMP-12 expression was reported to reside in the nucleus of human cardiomyocyte cells [70] and exogenously added recombinant MMP-12 protein, or its catalytic domain, observed to traffic to the nucleus, when used to treat MMP-12-silenced HeLa cells. Additionally, extracellular MMP-12 could cleave and inactivate systemic IFN-α, thereby attenuating the anti-viral inflammatory response as part of a negative feedback loop and which could be reversed upon treating virally-infected mice with the MMP-12 inhibitor, RXP470. Here, morbidity in mice was observed to be reduced, as was viral replication. Collectively, inhibition of extracellular MMP-12 or increasing its nuclear localization (or activity) highlights a potential basis on which the development of a therapeutic strategy against viral infection could be implemented.

The cellular anti-viral response against Dengue virus is augmented by MMP-3 [69]. Zuo et al. observed that the presence of nMMP-3 within infected cells was increased. The silencing of MMP-3 led to increased titers of the virus, decreased levels of cytokines and chemokines, and the reduced activity of NF-κB. Since nMMP-3 was found to be co-localized with intracellular NF-κB, it was suggested that the protease up-regulated the activity of NF-κB via a direct protein-protein interaction, which could subsequently promote the transcription of anti-viral and pro-inflammatory genes [69]. Collectively, such findings suggest that nMMP-3 plays a significant role in the anti-viral defense of the body against Dengue virus.

The requirement for MMPs for the immune response and protection of the organism against various infections has been suggested previously [110]. Predominantly, extracellular MMPs regulate the migration of immune cells, proteolysis of the basement membrane and the remodeling of the extracellular matrix. In the nucleus, MMPs are emerging to participate in the regulation of gene expression and regulate immunity against viruses and bacteria [69,70,74,111], but their additional abilities to cleave gene products that are central to negatively regulating the immune response cannot be completely excluded at this juncture.

## 8. Future Directions

MMPs were first identified in 1962 and since then have been characterized as extracellular proteases [112] which are firmly established as playing critical roles in oncogenesis and other pathological processes [113]. Over the years, MMPs have been understood as being some of the most pursued targets for drug development [9]. Their large family size, redundant roles, and substrate specificity are good reasons for why side effects arising from targeting them during disease progression with novel therapeutics has been a major obstacle for good therapeutics reaching the clinic. However, the detection of MMPs within nucleus, when taken with the biological effects they regulate, raises renewed optimism for developing therapeutics that are specific for these MMP derivatives. Mechanistically, the translocation of MMPs to the nucleus have only been thoroughly investigated for MMP-3 [58]. While bioinformatics approaches enable the identification of putative NLSs in other MMPs, their ability to translocate the proteases into nucleus and the mechanisms they utilize to do this still remain to be unveiled [61]. Such investigations would help in defining the plausibility of targeting nMMPs with the foresight of minimizing unwanted side effects, with greater clarity. The activity of MMPs can also be regulated by TIMPs [19]. So far, only TIMP1 has been reported within nuclei and no mechanism of its translocation has been described [25]. Since TIMPs also inhibit other a disintegrin and metalloproteinases (ADAMs), such as ADAM-10, therapeutic targeting nTIMP (so that nMMPs can take greater effect) may offer limitations [114].

A number of approaches have been adopted with a view to targeting MMPs for therapeutic purposes. For example, the small-molecule inhibitors hydroxamic acid, carboxylic acid, 5,5-disubstituted barbiturates, benzosulfonamide, and phosphonate have all shown efficacy in reducing oncogenesis, but unwanted side-effects have presented a number of challenges [115]. Alternatively, the more specific approach of targeting metalloproteases using single chain antibody fragments (scFV) has shown some encouraging outcomes in vitro [116,117,118]. Similarly, scFv fragments developed against extracellular MMP-14 have also shown good efficacy against cancer cell invasiveness in cell line models validated in a mouse orthotopic xenograft model [118]. Moreover, monoclonal antibodies directed at MMP-14 also successfully prevented the activation of pro-MMP-2 while antibodies to MMP-9 interfered with the catabolism of gelatin [119]. Collectively, while such approaches do encouragingly highlight the feasibility of targeting extracellular MMPs, their ability to target nMMPs remain to be explored.

For effective nMMP-specific inhibitor design, it may be necessary to elucidate the biological functions of distinct nMMPs and their mechanisms utilized for nuclear translocation. Since some of the proteases play important physiological and biological roles, as in the immune and anti-viral response, while others even suppress tumor growth via apoptosis, inhibiting nMMPs may require careful consideration that would potentially leave otherwise favorable biological effects intact [56,66,69,70]. Nevertheless, one serious challenge in targeting nMMPs is the specific delivery of the inhibitor to the nucleus and over the last ten years, several nano-carriers have been developed to help overcome this potential obstacle [120]. Such carriers have proven their efficiency in drug-targeting approaches for human cervical cancer, human oral squamous carcinoma cell lines, and multidrug-resistant breast cancer cell lines and in vivo, using MCF-7-derived breast tumor-bearing mice [121,122,123,124]. Alternatively, the use of such carrier systems with MMP inhibitors in combination with other conventional therapeutic reagents may have some usefulness to help combat tumor development, migration and metastasis potential [125,126,127,128].

## 9. Conclusions

Extracellular MMPs regulate a variety of functions, such as the development of tissues, inflammation, apoptosis, migration, angiogenesis, vasculogenesis, and other processes. However, it is emerging that nuclear matrix metalloproteinases are functionally distinct, through them performing unique and mutually exclusive functions within the nucleus. Although almost 25 years have passed since the first reports of nuclear localization of MMPs appeared, much remains to be explored. Here, one of these key areas is how the matrix metalloproteinases, whether secreted or anchored in the cell membrane, are transported to the cell’s nucleus. While some MMPs encode a classical NLS, others are transported through endocytosis. The activity of nMMPs is diverse in that they can promote tumor metastasis and other pathological processes. While on the one hand, nMMPs can contribute to apoptosis resulting in tumor cell death, on the other hand, nMMPs can positively regulate the immune response towards viral and bacterial infections. Surprisingly, the MMP inhibitor TIMP1 has also been detected within the nucleus and its full repertoire of inhibitory functions remains to be fully elucidated, in addition to whether other TIMPs can also reside in the nucleus.

Depending on the favorable or unfavorable effects of nMMP proteins, there appears to be some flexibility presented in how nMMPs may be targeted based on the manner in which they mechanistically translocate to the nucleus. For example, one can try to respectively elevate or interrupt nuclear transport through targeting nTIMP-chaperone effects or directly targeting the nMMPs in a ”compartment-specific” manner as the proteases traffic to the nucleus. To create specific targeting approaches for nMMPs activities, it is necessary to understand the biochemical network of these proteases in detail and gain a greater understanding of what other key biological effects these proteases may be regulating during disease progression, as a fundamental prerequisite.

## Figures and Tables

**Table 1:** Functions and localization of nuclear MMPs (nMMP) and nuclear TIMP (nTIMP). The table represents nMMPs and nTIMP1 and their functions in different cells and tissues. Malignant cells and tissues are indicated in red. Other pathological conditions are indicated in purple; a-deoxyribonucleic acid.

nMMP/nTIMP	Function	Cell Line or Tissue Type	Ref.
MMP-1	Apoptosis ↓	Human Muller glia	[75]
	Carcinogenesis ↑	Human breast cancer	[76]
	Not defined	Human keratinocytes, gingival tissue, megakaryocytes	[67,77,78]
MMP-2	Blood-brain barrier ↓	Mouse brain	[65]
	DNA^a^ reparation ↓	Human mesothelioma, cardiac myocytes; rat liver; pig pulmonary artery endothelial cells	[34,55,79]
	DNA reparation ↓ Apoptosis ↑	Rat brain neurons	[80,81]
	Carcinogenesis ↑	Human hepatocellular carcinoma	[33]
	Muscle adaptation to training ↑	Rat skeletal muscle fibers	[82]
	Not defined	Human melanoma cells, cutaneous squamous cell carcinoma, actinic keratosis, normal skin, megakaryocytes, endothelial cells; rat neurons; mouse skeletal muscle fibers	[25,72,78,83,84,85]
MMP-3	Apoptosis ↑	Human hepatocellular carcinoma, hepatocellular carcinoma cell line, peritumoral liver, liver myofibroblasts; Chinese hamster ovary cells	[56,66]
	Cell migration ↑	Human normal, osteoarthritic chondrocytes	[58]
	Immune response ↑	Human embryonic kidney epithelial cell line, macrophages	[69]
	Not defined	Human megakaryocytes	[78]
MMP-7	Cell migration and wound healing ↑	Human prostate cancer cell lines; mouse prostate tumor	[68]
	Not defined	Human adenocarcinoma, condyloma, normal squamous, columnar epithelium	[73]
MMP-9	DNA reparation ↓ Apoptosis ↑	Rat brain neurons	[80,81]
	DNA reparation ↓	Human epithelioid mesothelioma cell line	[34]
	Osteoclastogenesis ↑	Mouse preosteoclasts	[86]
	Not defined	Human tubular atrophic renal tubules, gingival tissue, megakaryocytes; dog neuropil and neurons	[77,78,87,88]
MMP-12	Immune response ↑	Human cervical cancer cell line, myocardial cells, bronchial epithelial cell line, mouse fibroblasts, cardiomyocytes cell line	[70,89]
MMP-13	Carcinogenesis ↑	Human oral tongue squamous cell carcinoma	[36,90]
	Not defined	Human chondrosarcoma of the jaws, brain tissues; rat brain tissues, chondrocytes	[15,32,91]
MMP-14	Carcinogenesis ↑	Human hepatocellular carcinoma, hepatocellular carcinoma cell line	[33]
	Immune response ↑	Mouse bone marrow-derived macrophages	[74]
MMP-16	Not defined	Human adenocarcinoma, condyloma, normal squamous, columnar epithelium	[73]
TIMP1	Cell growth ↑	Human gingival fibroblasts cell line	[23]
	Not defined	Human breast carcinoma cell line, endothelial cells; rat neurons	[24,25]
